# Development and Evaluation of a Retrieval-Augmented Generation Chatbot for Orthopedic and Trauma Surgery Patient Education: Mixed-Methods Study

**DOI:** 10.2196/75262

**Published:** 2025-10-23

**Authors:** David Baur, Jörg Ansorg, Christoph-Eckhard Heyde, Anna Voelker

**Affiliations:** 1 Department for Orthopedics, Trauma and Plastic Surgery, University Hospital Leipzig, Liebigstraße 20, Leipzig, Saxony, 04103, Germany, 49 3419723000, 49 3419723009; 2 Professional Association for Orthopaedics and Trauma Surgery (BVOU), Berlin, Germany

**Keywords:** retrieval-augmented generation, RAG, orthopedic patient education, medical chatbots, artificial intelligence in healthcare, large language models, LLM, clinical decision support systems, natural language processing, NLP, health information retrieval

## Abstract

**Background:**

Large language models are increasingly applied in health care for documentation, patient education, and clinical decision support. However, their factual reliability can be compromised by hallucinations and a lack of source traceability. Retrieval-augmented generation (RAG) enhances response accuracy by combining generative models with document retrieval mechanisms. While promising in medical contexts, RAG-based systems remain underexplored in orthopedic and trauma surgery patient education, particularly in non-English settings.

**Objective:**

This study aimed to develop and evaluate a RAG-based chatbot that provides German-language, evidence-based information on common orthopedic conditions. We assessed the system’s performance in terms of response accuracy, contextual precision, and alignment with retrieved sources. In addition, we examined user satisfaction, usability, and perceived trustworthiness.

**Methods:**

The chatbot integrated OpenAI’s GPT language model with a Qdrant vector database for semantic search. Its corpus consisted of 899 curated German-language documents, including national orthopedic guidelines and patient education content from the Orthinform platform of the German Society of Orthopedics and Trauma Surgery. After preprocessing, the data were segmented into 18,197 retrievable chunks. Evaluation occurred in two phases: (1) human validation by 30 participants (orthopedic specialists, medical students, and nonmedical users), who rated 12 standardized chatbot responses using a 5-point Likert scale, and (2) automated evaluation of 100 synthetic queries using the Retrieval-Augmented Generation Assessment Scale, measuring answer relevancy, contextual precision, and faithfulness. A permanent disclaimer indicated that the chatbot provides general information only and is not intended for diagnosis or treatment decisions.

**Results:**

Human ratings indicated high perceived quality for accuracy (mean 4.55, SD 0.45), helpfulness (mean 4.61, SD 0.57), ease of use (mean 4.90, SD 0.30), and clarity (mean 4.77, SD 0.43), while trust scored slightly lower (mean 4.23, SD 0.56). Retrieval-Augmented Generation Assessment Scale evaluation confirmed strong technical performance for answer relevancy (mean 0.864, SD 0.223), contextual precision (mean 0.891, SD 0.201), and faithfulness (mean 0.853, SD 0.171). Performance was highest for knee and back-related topics and lower for hip-related queries (eg, gluteal tendinopathy), which showed elevated error rates in differential diagnosis.

**Conclusions:**

The chatbot demonstrated strong performance in delivering orthopedic patient education through an RAG framework. Its deployment on the national Orthinform platform has led to more than 9500 real-world user interactions, supporting its relevance and acceptance. Future improvements should focus on expanding domain coverage, enhancing retrieval precision, and integrating multimodal content and advanced RAG techniques to improve robustness and safety in patient-facing apps.

## Introduction

In recent years, machine learning, particularly through large language models (LLMs), has significantly influenced various industries, with profound implications for health care. These advanced models have transitioned from experimental frameworks to practical tools that enhance patient care and medical research. By analyzing complex, large-scale datasets, LLMs enable health care professionals to extract insights related to patient data, disease trends, and treatment efficacy [1]. Their expanding role includes apps in disease diagnosis, medical documentation, and patient-provider communication, improving the efficiency and quality of health care services.

A key advancement in LLMs is their ability to process and interpret extensive contextual information, a crucial feature in health care, where context profoundly affects diagnostic and therapeutic decisions. For instance, OpenAI’s GPT series has demonstrated improved proficiency in comprehending and generating complex medical dialogs and texts, supporting apps such as automated medical documentation and patient communication [2].

In diagnostics, LLMs assist in extracting and summarizing essential information from unstructured medical data, including clinical notes and imaging reports, facilitating timely and accurate condition assessments [3]. In addition, these models contribute to medical education by providing interactive learning platforms and access to vast medical literature, enhancing the training and preparedness of medical students and professionals [4].

Patient engagement is another critical area where LLMs are used. By delivering clear and informative explanations about medical conditions and treatments, these models enhance patient understanding and adherence to treatment plans [5]. However, their deployment in health care requires caution due to the potential for generating misleading or incorrect information, known as “hallucinations.” Such errors can result in misinformed decisions that may compromise patient safety [6].

To mitigate these risks, enhancing the reliability of LLM-generated information is essential. Techniques such as retrieval-augmented generation (RAG), which integrates the generative capabilities of LLMs with data retrieval from trusted sources, have been developed to improve accuracy and verifiability, thereby preserving the integrity of medical advice [7].

Providing patient information on symptoms and conditions in orthopedics and trauma surgery presents unique challenges, as treatment success often depends on patients’ understanding of their condition and adherence to therapeutic measures [8]. Traditional methods, including printed materials, online education, and brief personal consultations, have limitations in addressing the comprehensive informational needs of orthopedic patients [9]. Digital solutions are gaining traction for their ability to provide accessible, personalized, and on-demand information, with specialized orthopedic apps demonstrating improved patient outcomes through disease education, tailored rehabilitation programs, and real-time feedback [10].

Despite growing interest in RAG apps in health care, limited empirical research has evaluated such systems specifically within orthopedic settings involving both medical professionals and patients [7,11]. This study seeks to address this gap by developing and evaluating a RAG-enhanced chatbot designed to provide patient-centered information on orthopedic symptoms and conditions.

By integrating multiple evaluation approaches, this study contributes to the emerging literature on specialized RAG apps in health care, with implications for improving patient education in orthopedics and trauma surgery. The primary objective is to develop and evaluate a RAG chatbot that delivers evidence-based information on orthopedic conditions while comprehensively assessing its performance through feedback from both medical professionals and patients, as well as automated metrics. 

## Methods

### Dataset and Preprocessing

Our dataset comprised official guidelines, specialized medical literature, and curated articles licensed from the Berufsverband für Orthopädie und Unfallchirurgie (BVOU-Germany), including texts available on the Orthinform platform [12]. These materials encompassed expert-reviewed medical content and patient education resources, all in German.

To enable contextualized medical information retrieval, we developed an RAG framework using LangChain and OpenAI models. This framework ensures that generated responses are derived from relevant, authoritative sources, supporting an evidence-based and transparent methodology [13,14]. A preprocessing pipeline was implemented to normalize and structure the dataset for efficient retrieval. JSON documents were standardized by extracting and refining metadata, including document names, categories, page numbers, URLs, and timestamps. PDFs were processed using unstructured.partition.pdf, preserving document structure and hierarchical elements. PyMuPDF was used as a fallback when unstructured failed to extract content correctly. To improve text quality, regex-based normalization removed artifacts.

The text was segmented using RecursiveCharacterTextSplitter, with 1000-character chunks and 200-character overlaps. URLs embedded in the text were preserved, and HTML tags were intentionally retained to maintain the structural integrity of web-based content. To enhance traceability, each chunk was assigned a unique identifier generated via MD5 hashing, and the processed data were stored in JSONL format, optimized for retrieval-based workflows.

### Vectorization and Document Retrieval

To enable semantic search and efficient retrieval, we implemented Qdrant as a vector database and indexed documents using OpenAI’s text-embedding-3-large model. This facilitated high-dimensional vector representations, ensuring precise retrieval of semantically relevant medical texts.

The Qdrant collection was initialized with a 3072-dimensional vector space, using cosine similarity as the distance metric. Processed text chunks were embedded in batches of 50 to optimize performance. The resulting vectors, along with their associated metadata, were stored in Qdrant and efficiently upserted for retrieval [15].

### Chatbot Implementation and Query Processing

A Streamlit-based chat interface was developed to enable interactive, real-time retrieval of medical information. The system integrates OpenAI for response generation and Qdrant for document retrieval, supporting a conversational RAG framework. The retrieval mechanism is history-aware, dynamically adapting to previous interactions and retrieving up to 5 relevant document chunks per query to ensure contextually rich responses.

The retrieval pipeline operates with a predefined search prompt, optimizing precision in medical document retrieval to ensure alignment with evidence-based orthopedic and trauma surgery guidelines. Retrieved documents are then passed to a response generation chain, conditioning GPT-4o to structure answers based on medical system instructions and ongoing conversation context. While the underlying GPT-4o model supports more than 50 languages, the chatbot’s responses are primarily grounded in a curated German-language knowledge base (BVOU guidelines, patient education content). Therefore, while multilingual queries are technically possible, factual accuracy is optimized for German.

To ensure that all chatbot responses remain strictly grounded in the retrieved medical context and to prevent hallucinations, we implemented a structured system prompt written in German. This prompt explicitly instructs GPT-4o to generate answers exclusively based on the retrieved documents. If no relevant information is found in the retrieved content, the model is required to return a predefined message stating that sufficient information is not available. The system prompt includes the instruction:

Beantworte die folgende Frage basierend ausschließlich auf den Dir vorgelegten Kontext. Wenn du in dem Kontext der Dir vorgelegt wird keine Antwort auf die Frage findest, sag dem User: ‘Dazu liegen mir leider keine Informationen vor.’ Du darfst keine Empfehlungen geben, keine Diagnosen stellen, keine Inhalte erfinden oder spekulieren. Formuliere sachlich, klar und laienverständlich. Verwende nicht mehr als 250 Wörter, außer der User fordert ausdrücklich mehr. Verwende ausschließlich den folgenden Kontext: {context}

The English translation of the above instruction is given below for clarity:

Answer the following question based strictly on the provided context. If the context does not contain sufficient information to answer the question, respond to the user: ‘Unfortunately, I do not have any information on that.’ You are not allowed to make recommendations, provide diagnoses, invent content, or speculate. Be objective, clear, and use layperson-friendly language. Do not exceed 250 words unless explicitly requested. Use only the following context: {context}.

The placeholder {context} is dynamically replaced at runtime with the top-k (k=5) most relevant document chunks retrieved from the Qdrant vector database. These context segments are semantically matched to the user query and serve as the exclusive knowledge base from which the response is generated. This prompt configuration ensures that the model does not rely on its pretrained general knowledge or generate information beyond the curated orthopedic corpus, thereby minimizing the risk of hallucinated or unverifiable content.

To support manual validation and optimization, a debug interface was implemented to inspect retrieved documents, evaluate chunk segmentation, and analyze token consumption. A system prompt was designed to strictly limit response length (250 words, up to 1000 if explicitly requested) and ensure that the chatbot provides only factually grounded answers. The search prompt dynamically reformulates queries based on conversation history to enhance retrieval accuracy.

The chatbot streams artificial intelligence (AI)-generated responses dynamically, maintains a structured session history, and displays extracted URLs from retrieved documents to ensure source transparency and clinical accountability for users.

### User Study and Evaluation

To evaluate the chatbot’s clinical reliability and user acceptability, we conducted a structured user study via Google Forms, allowing participants to interact with the system and provide qualitative and quantitative feedback. Participants were instructed to input predefined orthopedic queries into the chatbot, covering topics such as herniated discs, hip osteoarthritis, anterior cruciate ligament injuries, and congenital muscular torticollis.

A total of 12 predefined orthopedic questions were developed by 2 independent board-certified orthopedic specialists to represent common clinical inquiries for each topic. The responses were rated using two key metrics on a 5-point Likert scale: (1) response accuracy measures how precisely the chatbot provided medically valid answers, and (2) helpfulness assesses how effectively the chatbot assisted users in understanding medical conditions and treatments.

For each question, mean (SD) values were calculated to quantify the consistency and reliability of ratings. Likert-scale data were treated as interval-level data for descriptive statistical analysis, which we considered acceptable due to the scale’s symmetric design and widespread use of mean values in user experience (UX) research.

Beyond response evaluation, the survey included structured sections on empathy, clarity, usability, and response time. Users rated the chatbot’s friendliness, ability to recognize intent, trustworthiness, and clarity of medical explanations. In addition, participants assessed navigation, response latency, and overall satisfaction. At the end of the survey, users were given the opportunity to provide open-ended feedback for further improvements. The test group included a mix of medical professionals (students, residents, and specialists) and nonmedical users to reflect diverse perspectives, but no subgroup analysis was conducted.

### Automated Evaluation Using Retrieval-Augmented Generation Assessment Scale

To supplement the user-based evaluation, we applied the Retrieval-Augmented Generation Assessment Scale (RAGAS) framework, focusing on three key automated performance metrics [16]: (1) answer relevancy measures how well the chatbot’s response aligns with the user query; (2) context precision assesses the quality and medical relevance of retrieved documents; and (3) faithfulness ensures that responses are grounded solely in retrieved medical contexts, preventing hallucinations.

We generated 100 synthetic test questions using GPT-4o, ensuring each question was grounded in retrieved medical contexts. Corresponding ground truth answers were synthesized and manually reviewed by 2 board-certified orthopedic and trauma surgeons to ensure clinical accuracy and guideline adherence. Any ambiguous or incorrect responses were corrected to align with evidence-based medicine and current best practices. The 100-question test set was processed through the RAG pipeline, retrieving documents via Qdrant and generating responses using GPT-4o. The RAGAS framework then scored each response against ground truth answers using the 3 core evaluation metrics. Evaluation was conducted in batches, and performance metrics were aggregated into a quantitative evaluation report. Heatmaps and statistical summaries were generated to visualize chatbot performance across key medical retrieval tasks. This approach ensured that:

1. Retrieved contexts were clinically relevant (context precision).
2. Responses remained factually grounded in retrieved medical literature (faithfulness).
3. Answers directly addressed the medical queries posed by users (answer relevancy).

By combining user-based evaluations with structured, automated performance assessments, we systematically validated the chatbot’s ability to retrieve, interpret, and generate medically accurate orthopedic and trauma surgery information.

### Synthetic Query Generation for RAGAS Evaluation

For the automated RAGAS evaluation, 100 synthetic test questions and corresponding ground truth answers were generated using GPT-4o, based exclusively on the curated orthopedic knowledge base. This process ensured that all test questions were clinically relevant, context-grounded, and reproducible Textboxes 1 and 2.

Textbox 1. **Prompt for question generation.**

Based on this medical context:

[context content]

Generate a specific and challenging patient question on the topic [topic] ([subtopic]) that:

1. Can be answered using the information from this context
2. Tests understanding of medical concepts
3. Requires precise information from the text
4. Could realistically be asked by a patient

The question should sound natural and specific.

Textbox 2. **Prompt for ground truth answer generation.**

Based on this medical context:

[context content]

Answer this patient question:

[question]

The answer should:

1. Use only information from the provided context
2. Be precise and complete
3. Be formulated in patient-friendly language
4. Be 3‐5 sentences long

### Ethical Considerations

The study was conducted in accordance with the Declaration of Helsinki (World Medical Association; latest version) and applicable national regulations. It used an anonymous, voluntary online questionnaire and did not collect personal identifiers, IP addresses, or contact details. Under the Statutes of the Ethics Committee of the Medical Faculty, Leipzig University (§1(3)), formal review is required when personal data are processed; our protocol analyzed only anonymous responses. Based on this institutional policy and General Data Protection Regulation Recital 26 (anonymous data are not personal data), no formal ethics review was required at our institution. Participation implied consent after reading an online information sheet [17, 18].

## Results

### Preprocessing and Data Preparation

A total of 899 unique documents were processed, encompassing structured and unstructured medical content from sources such as Orthinform.de, German orthopedic guidelines, and additional curated literature. The dataset was segmented and preprocessed to enable efficient retrieval and response generation within the RAG framework.

Following the preprocessing pipeline, 18,197 unique text chunks were generated and stored in the Qdrant vector database, optimizing retrieval. These chunks were embedded using OpenAI’s text-embedding-3-large model, facilitating high-dimensional semantic search across patient education materials. The dataset contained 5,565,958 tokens, with an average chunk size of 973.27 characters. The largest chunks measured 1000 characters, while the smallest segments were 143 characters, ensuring a structured yet comprehensive representation of the source materials.

### Human Validation

The user evaluation included 30 participants: 13 licensed physicians (43.3%), 3 resident physicians (10%), 7 medical students (23.3%), and 10 individuals without a medical background (33.3%). Participants rated chatbot responses to 12 predefined orthopedic queries using a 5-point Likert scale. The queries covered four main orthopedic conditions: a herniated disc, hip osteoarthritis (coxarthrosis), anterior cruciate ligament tear, and congenital muscular torticollis. For each condition, 3 specific subquestions addressed pathophysiology, symptoms, and treatment options. A homogeneous test group was not considered essential for the purposes of this study, as the primary focus was on evaluating UX, transparency, and response plausibility from different user perspectives. Including both medical and nonmedical participants allowed for a more realistic appraisal of trust, clarity, and usability in patient-facing contexts.

### Assessment of Disease-Specific Responses

The accuracy ratings of chatbot responses ranged from 4.41 to 4.65 (mean), while helpfulness ratings ranged from 4.48 to 4.74 (mean), Table 1. The highest accuracy rating (mean 4.65, SD 0.61) was recorded for the question on congenital muscular torticollis. The highest helpfulness rating (mean 4.74, SD 0.51) was observed for the question on hip osteoarthritis symptoms. SD values for accuracy ratings ranged from 0.49 to 0.81 and from 0.49 to 0.68 for helpfulness ratings. The highest variance in accuracy (SD 0.81) was measured for responses regarding herniated disc symptoms.

Across all responses, 93.2% received a rating of 4 or higher for accuracy and 95.8% for helpfulness. No systematically lower ratings were observed for treatment-related questions compared to pathophysiological or symptom-related queries.

Table 1 presents data from 30 users, summarizing the average ratings for the accuracy and helpfulness of chatbot responses to specific orthopedic questions. Values are reported as mean (SD). The ratings were collected using a 5-point Likert scale (1=very inaccurate and unhelpful, 5=very accurate and helpful). The data were collected during the evaluation phase in September 2024.

**Table 1.** Human validation of disease-specific questions from the chatbot.

Questions	How accurate was the information? mean (SD)	How helpful were the answers? mean (SD)
Herniated disc		
What exactly is a herniated disc?	4.45 (0.72)	4.58 (0.56)
What are the typical symptoms of a herniated disc?	4.41 (0.81)	4.60 (0.49)
What treatment options are available for a herniated disc and how do they differ?	4.52 (0.68)	4.48 (0.68)
Hip osteoarthritis		
What is coxarthrosis and how does this condition develop?	4.55 (0.57)	4.58 (0.56)
What symptoms are typical of hip osteoarthritis?	4.55 (0.62)	4.74 (0.51)
What treatments are available for coxarthrosis?	4.55 (0.67)	4.65 (0.61)
Anterior cruciate ligament tear		
What is an anterior cruciate ligament tear and how can it occur?	4.54 (0.62)	4.61 (0.56)
What symptoms indicate an anterior cruciate ligament tear?	4.61 (0.62)	4.68 (0.65)
What treatment options are available for an anterior cruciate ligament tear?	4.52 (0.68)	4.52 (0.63)
Congenital muscular torticollis		
What does congenital muscular torticollis mean and how can it be recognized in a baby?	4.65 (0.61)	4.61 (0.56)
What are the causes of congenital muscular torticollis?	4.61 (0.56)	4.65 (0.49)
What treatment options are available for babies with congenital muscular torticollis?	4.61 (0.49)	4.61 (0.56)

### User Experience

**Figure 1.**
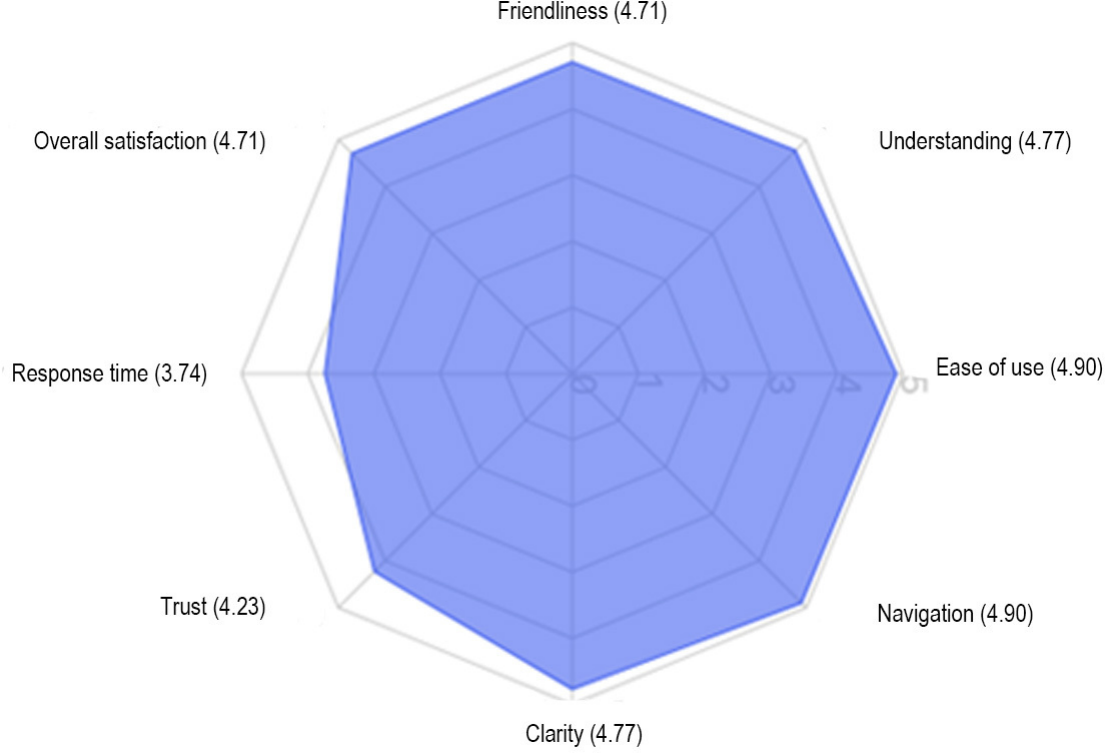
User experience evaluation of the orthopedic chatbot.

UX metrics were evaluated across 8 dimensions, as visualized in Figure 1. Ease of use and navigation intuitiveness received the highest ratings, both with mean values of 4.90 (SD 0.30). Response clarity (mean 4.77, SD 0.43) and understanding of user concerns (mean 4.77, SD 0.43) were also rated positively. Trust in the provided information was rated moderately lower (mean 4.23, SD 0.56). System response time received the lowest rating (mean 3.74, SD 0.73) and exhibited the highest variance. This may partly reflect limitations of the test environment: during the user evaluation, the chatbot was accessed via a local Streamlit app, which lacked the server-side performance optimization of the final production system. In contrast, the deployed version on the Orthinform platform demonstrates faster and more stable response times in real-world use. Overall satisfaction with the chatbot was high (mean 4.71, SD 0.53).

UX evaluation of the orthopedic chatbot during the user study (September 2024). The radar chart summarizes ratings across 8 dimensions (ease of use, navigation, response clarity, understanding of user concerns, trust, empathy, system response time, and overall satisfaction). Data were collected from 30 participants (licensed physicians, medical students, and nonmedical users) using a 5-point Likert scale (1=very poor and 5=excellent).

### Automated Evaluation Using RAGAS

The 100-question synthetic test set was processed through the chatbot’s retrieval pipeline, retrieving up to 5 (k=5) document chunks per query and generating responses using GPT-4o. RAGAS evaluation was conducted on 3 core metrics, with results reported as mean (SD) scores to quantify both performance and variability across the test set.

#### Answer Relevancy

The chatbot’s ability to generate responses directly aligned with user queries achieved a mean score of 0.864 ( SD 0.223). Queries related to back pain and shoulder problems scored the highest, with means of 0.917 and 0.882, respectively, while hip-related topics exhibited slightly lower performance, with a mean of 0.784 (SD 0.338).

#### Context Precision

The retrieval mechanism returned highly relevant documents per query, achieving a mean precision score of 0.891 (SD 0.201). The highest precision was observed for knee-related queries, with a mean of 0.959 (SD 0.078), while hip-related topics showed lower retrieval performance, with a mean of 0.809 (SD 0.362).

#### Faithfulness

Responses remained strongly grounded in retrieved documents, with a mean faithfulness score of 0.853 (SD 0.171). A total of 71% of responses were fully based on the retrieved context (scoring above 0.8), while 19% displayed minor inconsistencies or additional inferred information (scoring between 0.6 and 0.8).

**Figure 2.**
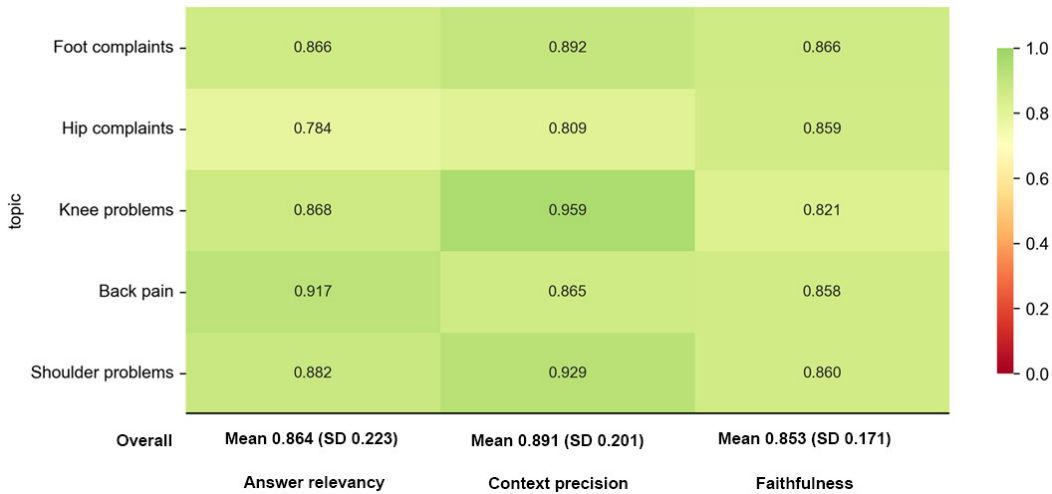
Retrieval-Augmented Generation Assessment Scale evaluation metrics across major orthopedic domains.

As shown in Figure 2, the chatbot demonstrates strong performance across all orthopedic domains, with most metrics scoring above 0.8. Notable performance variations were observed across different orthopedic topics. Knee-related queries showed the highest overall context precision score (0.959), indicating exceptionally accurate retrieval of relevant information. Back pain queries achieved the highest answer relevance (0.917), suggesting the system excels at generating targeted responses for this common musculoskeletal complaint.

Hip-related queries, while still performing adequately, showed comparatively lower scores across all 3 dimensions, particularly in answer relevancy (0.784). This indicates an area for potential improvement in the knowledge base or response generation for hip-related orthopedic conditions.

RAGAS evaluation metrics across major orthopedic domains (automated evaluation, October 2024). The chart visualizes answer relevancy, context precision, and faithfulness for 5 major orthopedic domains (back pain, hip, knee, shoulder, and foot problems) based on a synthetic test set of 100 GPT-generated queries. Values range from 0 to 1, with a color gradient from red (poor performance) to dark green (excellent performance). Overall mean (SD) values for each metric are displayed at the bottom of the chart.

### Performance Analysis and Observations

The subanalysis of the RAGAS evaluation of 100 synthetic test questions across 31 subcategories of major orthopedic conditions reevaluated the chatbot’s performance using 3 key metrics: answer relevancy, context precision, and faithfulness. A comprehensive analysis demonstrates that 86% of all queries attained values above 0.8 in both answer relevancy and context precision, while 92% of responses exhibited answer relevancy values above 0.8. Among the 6% of queries with answer relevancy values below 0.1, 60% pertained to the hip domain (primarily tendinitis), 25% to foot complaints, and the remaining 15% to various other conditions.

**Figure 3.**
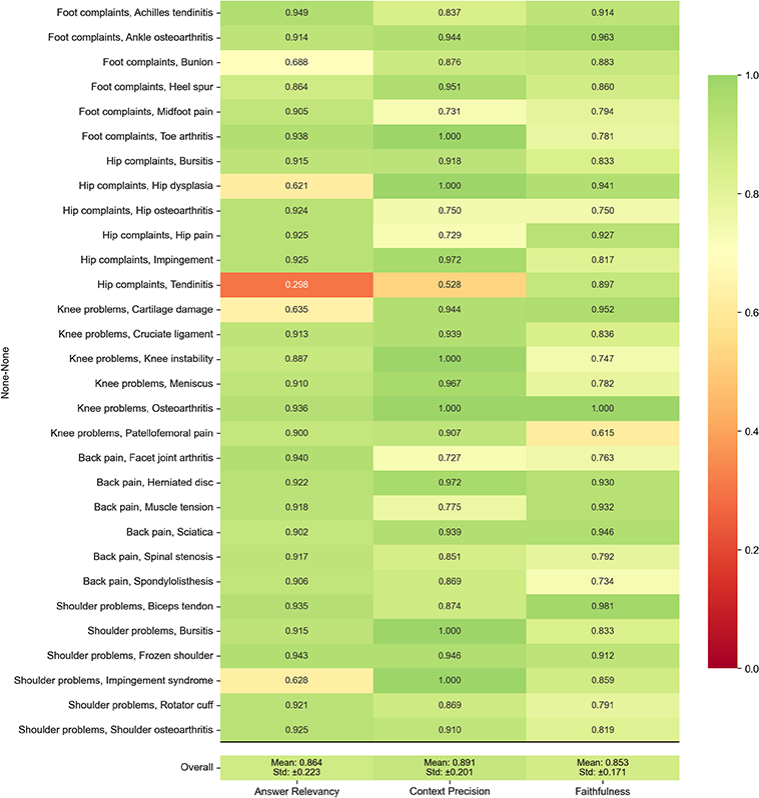
Retrieval-Augmented Generation Assessment Scale performance metrics across 31 orthopedic subtopics.

Figure 3 illustrates performance metrics across all 31 orthopedic conditions examined. Several subtopics reached the maximum score (1) in individual metrics. In context precision, this applied to toe arthritis, hip dysplasia, knee instability, shoulder bursitis, and shoulder impingement syndrome. Knee osteoarthritis achieved the maximum value of 1 in faithfulness, combined with high answer relevancy (0.936) and maximum context precision (1). In contrast, hip tendinitis exhibited the lowest answer relevancy value of 0.298 in the entire dataset.

RAGAS performance metrics across 31 orthopedic subtopics (automated evaluation, October 2024). The chart visualizes answer relevancy, context precision, and faithfulness across 31 specific orthopedic subcategories, grouped by their main domains. Values range from 0 to 1 and are displayed using a color gradient from red (poor performance) to dark green (excellent performance). Overall, mean (SD) values for each metric are provided at the bottom of the chart.

A detailed analysis of individual questions accounts for the performance deficits in hip tendinitis, as illustrated in Figure 2. Out of the 3 queries on this topic, 2 (Q19: differential diagnosis of tendinitis; Q20: clinical distinguishing features) received answer relevancy values of 0, while the third query (Q18: therapeutic options) achieved 0.893. This uneven distribution resulted in a low average value of 0.298.

Overall, 3% of the 100 test questions exhibited total context retrieval failures (score=0). Hip-related topics were disproportionately affected, with complete failures in 15% of answer relevancy assessments and 20% of context precision assessments. These failures primarily occurred in complex differentiation tasks, notably Q19 (hip tendinopathy) and Q6 (hip impingement syndrome).

### Qualitative Output Analysis and Sample Responses

To illustrate the output quality of our system, we selected representative user queries along with the corresponding retrieved passages, generated answers, and associated evaluation metrics (answer relevancy, context precision, and faithfulness). These examples reflect typical dialog scenarios and demonstrate both the content quality and the traceability of source references. The selected samples cover a range of anatomical regions (eg, lumbar spine, knee joint, and shoulder) and include questions on diagnostics, conservative management, and surgical decision-making.

Table 2 presents 2 representative examples of user queries, retrieved context excerpts, and corresponding RAGAS evaluation scores from the automated test set. The user queries and 2 sample answers have been translated from German into English, while other context excerpts remain in the original language to ensure fidelity. All examples are based on the synthetic evaluation conducted since October 2024. A complete list of all original queries, answers, and evaluation metrics is provided in MultimediaAppendices 1  2.

**Table 2.** Representative examples of user queries, retrieved context excerpts, and Retrieval-Augmented Generation Assessment Scale evaluation scores.

User query	Retrieved context (excerpt)	Answer relevancy	Context precision	Faithfulness
What conservative treatments are typically used for a lumbar disc herniation, and under what circumstances is surgery unavoidable?	Conservative treatments include physiotherapy, medication, targeted spinal injections, weight reduction, and avoiding prolonged sitting. Surgery is considered if symptoms persist for more than 6 months despite therapy or in cases of neurological deficits or loss of bladder/bowel control.	0.974	1	1
What is the most common location for cartilage damage in the knee joint detected in MRI^a^ examinations with a field strength of 3.5 Tesla, and how do the results of these examinations, together with pain localization and intensity, influence the selection of therapy?	In MRI examinations of the knee joint with a field strength of 3.5 Tesla, the most common location for detecting cartilage damage is the medial femoral condyle, followed by the posterior surface of the patella, although the latter is much less common. The results of these MRI examinations, in conjunction with the patient’s clinical examination which includes pain localization and intensity, play a crucial role in influencing the selection of a therapy. While clinical tests rarely provide direct evidence of cartilage damage, the information gathered from them, alongside MRI and X-ray diagnostics, helps determine the appropriate treatment method. The key factors for selecting the therapy are the depth and extent of the cartilage defect that needs to be treated.	0.9702	0.833	1

a MRI: magnetic resonance imaging.

## Discussion

### Principal Results

The evaluation of our RAG chatbot for orthopedic and trauma surgery patient education demonstrated strong performance metrics. Human assessments yielded accuracy ratings averaging 4.55 (out of 5) and helpfulness ratings of 4.61 (out of 5) across orthopedic conditions. UX metrics showed high scores for ease of use (4.90) and response clarity (4.77).

### Comparison With Prior Works

Compared to previous studies on health care chatbots, our system demonstrates significantly higher performance in terms of accuracy, helpfulness, and user satisfaction. For instance, Nadarzynski et al [19] reported acceptance rates of 67% and trust scores averaging 3.4 out of 5 in a general population sample, reflecting persistent concerns about reliability and factual correctness. Similarly, Milne-Ives et al [20] observed usability scores typically ranging between 3.5 and 4.2 in various chatbot apps, with considerable heterogeneity depending on system design and target audience. In contrast, our chatbot achieved mean scores of 4.55 for perceived accuracy and 4.61 for helpfulness, supported by strong ratings for usability and clarity. These differences may partly be explained by the academic background of our user cohort, but they also reflect domain-specific design choices and system robustness.

A notable strength of our study lies in the integration of human and automated evaluation strategies. Beyond structured user feedback, we applied the Retrieval-Augmented Generation Assessment Score (RAGAS) framework to evaluate the chatbot’s contextual precision, answer relevancy, and grounding fidelity. The chatbot achieved strong mean scores across all 3 dimensions (eg, answer relevancy: mean 0.864, SD 0.223), indicating effective retrieval and factual consistency. The relatively high SD values reflect expected variability across subdomains, consistent with known topic-specific limitations in medical LLM performance.

In direct comparison to baseline LLMs without retrieval mechanisms, the advantages of RAG become evident. For example, Deng et al [21] showed that ChatGPT-4 provided accurate responses to only 17% of treatment-related orthopedic questions, while 75% were deemed merely comprehensive without being verifiably correct. In contrast, our system achieved 92% of responses above 0.8 in answer relevancy, demonstrating substantial improvements in factual alignment and contextual precision. These results align with findings by Jabal et al [22], who observed that retrieval-enhanced LLMs outperformed traditional models in structured data extraction tasks in clinical settings.

Beyond performance metrics, our system introduces several architectural and contextual innovations that distinguish it from existing chatbot solutions.

This domain-specific adaptation approach is comparable to methodologies applied in other specialized RAG contexts, such as religious text retrieval [23].

Recent studies further support this pattern: a 2-layer RAG architecture improved grounding and answer quality in medical Q&A, and RAG variants outperformed GPT-4 in clinical fact-checking, underscoring the value of retrieval for verifiable, source-linked outputs [24,25].

Unlike general-purpose medical chatbots that often rely solely on pretrained LLMs, our framework combines semantic retrieval with domain-specific grounding. All responses are generated based on verified German-language materials, including guideline-compliant content licensed from the BVOU. This ensures high clinical relevance and alignment with local standards of care.

Furthermore, the system is explicitly tailored to German-speaking users and reflects orthopedics-specific terminology, diagnostic reasoning, and patient communication norms. Unlike many tools focused on English-speaking audiences, our chatbot addresses the information needs of a specific linguistic and clinical population. The RAG architecture also promotes transparency by displaying the source URLs and retrieved text segments for each response, enabling traceability and user trust. Finally, our validation approach integrates structured Likert-based user ratings with RAGAS metrics and expert-reviewed reference answers, providing a robust multiperspective evaluation framework.

In summary, the combination of retrieval-based grounding, language and domain specificity, and transparent validation distinguishes our chatbot from conventional medical AI tools and supports its use in clinically relevant patient education scenarios.

### Real-World Implementation

Following the evaluation phase, the chatbot was integrated into the public patient education platform of the German Society of Orthopedics and Trauma Surgery (BVOU) and has been accessible via Orthinform.de since October 2024. This real-world deployment enables patients across Germany to access AI-guided orthopedic information around the clock. The system is clearly labeled as an informational tool, not intended for diagnosis, treatment decisions, or appointment scheduling. It does not support direct communication with clinics.

As of June 2025, the chatbot has processed a total of 9514 user interactions. The most frequent topics, in descending order of frequency, included back pain, hip joint degeneration, meniscus tear, knee pain, spinal stenosis, shoulder impingement, and disc herniation. These usage statistics underscore the chatbot’s practical relevance and indicate a high level of user acceptance for AI-assisted patient education in orthopedics.

### Limitations

Despite the overall strong performance, several limitations must be noted. First, hip-related queries showed disproportionately high failure rates, with 15% resulting in complete answer relevancy failures (score=0) and 20% exhibiting context precision failures. Questions involving complex differential diagnoses, such as distinguishing gluteal tendinopathy from trochanteric bursitis, proved particularly challenging for the retrieval system. This pattern of domain-specific performance variance, reflected in elevated SD values across metrics, suggests uneven coverage in the knowledge base or suboptimal document chunking in specialized orthopedic subdomains. These findings are consistent with observations by Johnson et al [26], who reported similar retrieval limitations in RAG-based diagnostic systems due to gaps in knowledge coverage.

Second, while the system performed reliably on standard diagnostic and treatment questions, more nuanced or comparative queries, such as those related to treatment efficacy or surgical decision-making, exhibited greater variability in faithfulness scores. The observed mean value of 0.853 (SD 0.171) in faithfulness suggests inconsistent grounding across different query types. Genovese et al [27] similarly reported that RAG model accuracy in patient education depends heavily on the depth and quality of the underlying dataset.

Third, there is a persistent risk of hallucinations. Although the retrieval architecture significantly reduces the likelihood of fabricated responses compared to standard LLMs, it cannot eliminate them entirely. The quality of generated outputs is directly tied to the relevance and completeness of the underlying documents. Underrepresented orthopedic subdomains may thus produce lower fidelity outputs. This aligns with concerns raised by Altofer et al [28], who described generative AI as a “double-edged sword” in medicine—capable of exceeding expert performance in some domains, yet also prone to generating misleading content.

Fourth, the current implementation omits several advanced RAG features that may enhance response quality. These include hybrid retrieval methods (combining sparse and dense search), contrastive answer generation, knowledge graph integration, and multimodal capabilities (eg, linking imaging findings with textual explanations). Future work should explore these approaches to strengthen context matching, improve factual precision, and reduce hallucination risks in complex query types.

Finally, a deliberately implemented safeguard in the form of a persistent disclaimer emphasizes that the chatbot serves solely for general informational purposes and is not intended for self-diagnosis or clinical decision-making. This ethical design feature underlines the system’s patient-centered focus and risk awareness. Future versions may benefit from the integration of an autonomous detection mechanism to recognize when a query exceeds the system’s informational boundaries and to proactively prompt users to consult a qualified health care professional.

Another promising enhancement is domain-specific knowledge organization. The observed regional performance variations, with knee-related queries outperforming hip-related ones, suggest that tailored knowledge representation strategies may be needed for different orthopedic subdomains. Introducing hierarchical retrieval mechanisms that model anatomical and pathological relationships could improve response accuracy for conditions requiring cross-document integration.

### Future Work

To address these limitations, advanced RAG mechanisms could further enhance the system’s capabilities. Implementing agentic RAGs, which dynamically reformulate queries based on conversational context, could improve performance on complex differential diagnostic questions. Unlike static top-k retrieval, this approach enables iterative, context-aware information gathering, particularly benefiting challenging hip-related queries identified in our evaluation.

In future work, domain-specific knowledge organization should also be explored. The observed regional performance variations, with knee-related queries outperforming hip-related ones, suggest that tailored knowledge representation strategies may be needed for different orthopedic subdomains. Introducing hierarchical retrieval mechanisms that model anatomical and pathological relationships could improve response accuracy for conditions requiring cross-document integration.

### Conclusions

Our RAG-based chatbot demonstrates strong potential for improving patient access to orthopedic information, with solid evaluation scores and reliable performance. While it already provides valuable support for patient education, continuous adaptation is essential to keep pace with rapid AI advancements and ensure optimal accuracy. Our data show strong overall performance with identifiable outliers where the RAG pipeline is less robust. To ensure consistent quality in patient education and broader medical use cases, performance should be validated across additional datasets and settings with clear, reproducible quality control. The impact of RAG will hinge on high-quality, well-managed corpora and reliable retrieval that keeps answers traceable to sources.

## Supplementary material

10.2196/75262 Multimedia Appendix 1 Complete performance of retrieval-augmented generation metrics with exact question formulations.

10.2196/75262 Multimedia Appendix 2 German synthetic data set with 100 questions and answers.

## Abbreviations

AI: artificial intelligence
BVOU: Berufsverband für Orthopädie und Unfallchirurgie
LLM: large language model
RAG: retrieval-augmented generation
RAGAS: Retrieval-Augmented Generation Assessment Scale
UX: user experience
